# Editorial: Translation of health technology reassessment and its outputs of disinvestment, de-implementation and de-adoption of drugs and medical devices in practice-what have we learned?

**DOI:** 10.3389/fphar.2025.1700196

**Published:** 2025-10-13

**Authors:** Rosmin Esmail, Nora Ibargoyen-Roteta, Maximilian Otte, Iñaki Gutiérrez-Ibarluzea, Roberta Joppi, Hans-Peter Dauben

**Affiliations:** ^1^ Acute Care Alberta, Calgary, AB, Canada; ^2^ Department of Community Health Sciences, University of Calgary, Calgary, AB, Canada; ^3^ Department of Medicine, University of Alberta, Edmonton, AB, Canada; ^4^ O’Brien Institute for Public Health, Calgary, AB, Canada; ^5^ Basque Office for HTA (Osteba), Basque Foundation for Health Innovation and Research (BIOEF), Bilbao, Spain; ^6^ EuroScan International Network e. V., Cologne, Germany; ^7^ Institut für Gesundheit und Gesellschaft (I2GG), Cologne, Germany; ^8^ Directorate of Health Research, Innovation and Evaluation, Ministry for Health, Basque Government, Vitoria-Gasteiz, Spain; ^9^ Pharmaceutical Department - Local Health Authority of Verona, Verona, Italy

**Keywords:** disinvestment, deadoption, deimplementation, knowledge translation, health technology reassessment, health technology assessment

## Introduction

Health technology assessment (HTA) has long been associated with the adoption and evaluation of innovations and new technologies. The current HTA definition includes the concept of ‘determining the value of a health technology at different points during its lifecycle’ (O'Rourke et al., 2020). Furthermore, healthcare and its sustainability depend on the ability to reassess technologies throughout their lifecycle to ensure they are being used optimally. Health Technology Reassessment (HTR) has gained increasing attention as a tool to re-evaluate technologies that are currently being used in healthcare systems. HTR is defined as “a structured, evidence-based assessment of the clinical, social, ethical, and economic effects of a technology, currently used in the healthcare system, to inform the optimal use of that technology in comparison to its alternatives” (Noseworthy and Clement, 2012). Reassessment could lead to an increased use or adoption, no change, decreased use, de-adoption^1^, disinvestment^2^, or de-implementation^3^ (Esmail et al., 2018). However, the implementation of the recommendations from a HTR process requires regulatory, cultural, political, ethical, and real-world evidence to succeed. In this editorial, we reflect on five case studies and highlight lessons learned to illuminate HTR’s achievements and persistent challenges.

## Regulation as a foundation for safe practice

One lesson is that regulatory frameworks shape what reaches patients. The study of Ala®sil, an ophthalmic irrigation solution withdrawn from the European market in 2016, demonstrated how insufficient scrutiny under the Medical Devices Directive (MDD) allowed a product with limited safety data to be marketed (Andrés-Iglesias et al.). Under the current Medical Devices Regulation (MDR 2017/745), such a device would not have been authorized. This example underlines how regulatory evolution enables the proactive *de-adoption* of unsafe or low-value technologies before widespread use occurs. Moreover, this study highlights the need to have HTA activities occur in close collaboration with regulatory authorities to ensure synergies among organizations with diverse objectives.

## Natural disinvestment through innovation

Not all disinvestment requires a formal policy decision: sometimes it happens naturally. The history of trastuzumab in the Veneto Region of Italy is a clear example of this phenomenon (Becchetti et al.). With the introduction of biosimilars and new formulations, *per capita* costs decreased, and resources were reallocated. Regional authorities used HTR methodologies to monitor these changes, ensuring continuity of care while guiding spending. This study demonstrates how competition, if accompanied by structured oversight, can act as a mechanism for “natural disinvestment”, freeing resources for innovation.

## The cost of delayed reassessment

Delays in disinvestment or adoption can translate directly into worse outcomes, as depicted in the case of the use of rituximab in the treatment of follicular lymphoma. A 16-year nationwide cohort study in Brazil showed that patients treated with rituximab-containing regimens had better survival rates than those treated with older chemotherapy schemes. However, this study showed that limited public access to this treatment meant that thousands of patients were treated suboptimally (Azevedo et al.). Subsequent delays in the disinvestment of outdated therapies prevent the adoption of superior treatments that can save lives.

## Rare diseases and the challenge of optimization

In rare diseases, HTR faces challenges such as high treatment costs and variable outcomes. A comprehensive retrospective study of Gaucher disease in Brazil showed that enzyme replacement therapies improved survival; however, these benefits varied based on dosing adequacy and early treatment initiation (Borin et al.). Given the extraordinary costs of these therapies, reassessment does not necessarily mean withdrawal; rather, it means optimizing use and tailoring investment to maximize benefit. In such contexts, HTR becomes a tool for balancing sustainability with ethical obligations toward patients with rare conditions, ensuring they receive optimal treatment.

## Real-world evidence as a driver of reassessment

Randomized controlled trials remain essential, but real-world data are increasingly shaping reassessment. For example, a large observational study in China found that SGLT2 inhibitors significantly slowed kidney disease progression and reduced renal events compared with other glucose-lowering drugs (Xiao et al.). These findings provide a compelling rationale for the progressive de-implementation of less effective alternatives in favor of SGLT2 inhibitors and demonstrate how real-world evidence can accelerate the translation of trial results into practice.

## Key lessons

Across these diverse studies, several lessons emerge for HTR:

1. Regulation is needed. Unsafe practices need to be monitored and regulated to ensure they are meeting patient needs throughout the HTR process. Further information should be obtained on all the drugs approved with specific commitments on efficacy.
2. Room for innovation. Natural reassessment can occur if the system allows for competition and innovation.
3. Reassessment needs to be timely. Evidence alone is not enough: cultural, political, and/or financial barriers can delay change.
4. Reassessment is about optimization. It may result in withdrawal, substitution, dose optimization, or resource reallocation among its several outputs to ensure optimal use of a technology.
5. Real-world evidence can be a driver in HTR decisions. Using clinical trials as a complement can ground HTR decisions in everyday practice and within diverse populations.

## Moving Forward

The translation of HTR recommendations into practice requires consideration of implementation (Esmail et al., 2021). Health systems should embed reassessment cycles into HTA agencies, invest in infrastructure for continuous monitoring, and build implementation science capabilities. Importantly, we must also shift the culture of medicine to embrace reassessment as a process of learning and progress throughout the life cycle of a technology (Gutiérrez-Ibarluzea et al., 2017).

These five cases emphasize that HTR is not merely a technical process. It requires robust regulation, innovation, timely decision-making, optimization, coordination among disciplines, and cultural acceptance through real-world evidence. They illustrate the breadth of HTR’s importance and relevance in managing the finite and limited resources of today’s healthcare systems.

## Footnotes

1 Defined as: “[…] discontinuation of a clinical practice after it was previously adopted” (Niven et al., 2015).

2 Defined as “It relates to the processes of (partially or completely) withdrawing health resources from any existing health care practices, procedures, technologies or pharmaceuticals that are deemed to deliver little or no health gain for their cost, and thus are not efficient health resource allocations.” (Elshaug et al., 2007).

3 Defined as “reducing or stopping the use or delivery of services or practices that are ineffective, unproven, harmful, overused, or inappropriate” (Norton et al., 2017).
